# T Cell Activation Markers and African Mitochondrial DNA Haplogroups among Non-Hispanic Black Participants in AIDS Clinical Trials Group Study 384

**DOI:** 10.1371/journal.pone.0043803

**Published:** 2012-08-27

**Authors:** Todd Hulgan, Gregory K. Robbins, Spyros A. Kalams, David C. Samuels, Benjamin Grady, Robert Shafer, Deborah G. Murdock, Doug Selph, David W. Haas, Richard B. Pollard, AIDS Clinical Trials Group, Victor De Gruttola, Sally Snyder, Thomas Nevin, Carla Pettinelli, Michael Dube, Margaret Fischl, Robert Delaphna, Linda Gideon, Richard D’Aquila, Stefano Vella, Thomas Merigan, Martin Hirsch

**Affiliations:** Harvard School of Public Health; Social & Scientific Systems; National Institutes of Health; Indiana University; Miami University; Howard University; Frontier Science and Technology Foundation; Vanderbilt University; Istituto Superiore de Sanita; Stanford University; Harvard Medical School; 1 Vanderbilt University School of Medicine, Nashville, Tennessee, United States of America; 2 Massachusetts General Hospital/Harvard University, Boston, Massachusetts, United States of America; 3 Stanford University Medical Center, Stanford, California, United States of America; 4 University of California Davis, Sacramento, California, United States of America; New York University, United States of America

## Abstract

**Introduction:**

Mitochondrial function influences T cell dynamics and is affected by mitochondrial DNA (mtDNA) variation. We previously reported an association between African mtDNA haplogroup L2 and less robust CD4 cell recovery on antiretroviral therapy (ART) in non-Hispanic black ACTG 384 subjects. We explored whether additional T cell parameters in this cohort differed by mtDNA haplogroup.

**Methods:**

ACTG 384 randomized ART-naïve subjects to two different nucleoside regimens with efavirenz, nelfinavir, or both. CD4 and CD8 memory and activation markers were available at baseline and week 48 on most subjects. mtDNA sequencing was performed on whole blood DNA, and haplogroups were determined. We studied non-Hispanic black subjects with HIV RNA <400 copies/mL at week 48. Analyses included Wilcoxon ranksum test and linear regression.

**Results:**

Data from 104 subjects were included. Major African mtDNA haplogroups included L1 (N = 25), L2 (N = 31), and L3 (N = 32). Baseline age, HIV RNA, and CD4 cells did not differ between L2 and non-L2 haplogroups. Compared to non-L2 haplogroups, L2 subjects had lower baseline activated CD4 cells (median 12% vs. 17%; p = 0.03) and tended toward lower activated CD8 cells (41% vs. 47%; p = 0.06). At 48 weeks of ART, L2 subjects had smaller decreases in activated CD4 cells (−4% vs. −11%; p = 0.01), and smaller CD4 cell increases (+95 vs. +178; p = 0.002). In models adjusting for baseline age, CD4 cells, HIV RNA, and naïve-to-memory CD4 cell ratio, haplogroup L2 was associated with lower baseline (p = 0.04) and 48-week change in (p = 0.01) activated CD4 cells.

**Conclusions:**

Among ART-naïve non-Hispanic blacks, mtDNA haplogroup L2 was associated with baseline and 48-week change in T cell activation, and poorer CD4 cell recovery. These data suggest mtDNA variation may influence CD4 T cell dynamics by modulating T cell activation. Further study is needed to replicate these associations and identify mechanisms.

## Introduction

The CD4^+^ T-lymphocyte is the primary cellular target of HIV, and the absolute CD4^+^ T lymphocyte count is a reliable determinant of disease progression and opportunistic infection risk among HIV-infected persons. The CD4 count is also a major factor in the decision to initiate antiretroviral therapy (ART) in asymptomatic HIV-infected individuals [1]. After initiating ART, there is substantial interindividual variability in the rate and magnitude of CD4 recovery [2]. Many ART-treated patients (as many as 30%) fail to attain substantial increases in CD4 count [2]–[3], and poorer CD4 count responses on ART are associated with disease progression despite adequate virologic responses [2], [4]. Host genetic variation appears to play a role in CD4 count recovery. Studies have suggested possible associations between CD4 count recovery on ART and single nucleotide polymorphisms (SNPs) in chemokine receptor [5]–[6], human leukocyte antigen [7]–[8], cytokine [9], and apoptosis-related [10] genes. These results highlight the possibility that an important host factor that influences HIV-infected CD4 cell turnover is regulation of apoptosis [11]–[12].

T cell activation is a highly energy-dependent process that is a hallmark of chronic HIV-1 infection and is a predictor of CD4 T cell recovery on ART [13]–[17]. T cell activation is associated with cellular apoptosis [18]–[20] and can be attenuated with ART, but not completely or to the same degree in all persons [21]–[22]. Given that mitochondria play critical roles in energy production, oxidative stress, and apoptosis, their importance in cellular immune responses and T cell turnover seems apparent. Indeed, the importance of mitochondria-mediated, intrinsic apoptotic pathways in normal T cell homeostasis [23]–[24], and in dysregulated CD4 T cell recovery during HIV-1 infection [25] have been described. Other investigations have identified associations between T cell activation and apoptosis that influence CD4 T cell depletion during HIV-1 infection and are improved by ART [26]–[28].

Within mtDNA, combinations of SNPs allow for categorization of individuals into haplogroups [29]. Haplogroups and other mtDNA variants have been associated with differences in mitochondrial function [30]–[31], and the clinical relevance of haplogroups- and mtDNA variation in general- for disease risk is well described [32]–[35], Few studies have examined mitochondrial DNA (mtDNA) in T cell subsets in HIV-1-infected subjects in the absence of ART toxicity. One described decreased mtDNA quantity in activated (CD8>CD4) T cells compared with non-activated subsets from HIV-1 seroconverters [36]. In another recent study, loss of mtDNA that correlated with activation was seen in CD8 T cells of HIV-1-infected, ART-naïve subjects [37].

With respect to genotypic mtDNA variation, a study from the Multicenter AIDS Cohort Study (MACS) recently reported that mtDNA variants were associated with progression to AIDS and/or death in untreated, HIV-1-infected Caucasians [38]. We previously reported associations between several mtDNA SNPs- and with the major African mtDNA haplogroup L2 defined by several of these SNPs- and magnitude of CD4 T cell recovery after ART initiation in non-Hispanic black participants in AIDS Clinical Trials Group (ACTG) study 384 [39]. It is plausible that functional variation in mtDNA would influence T cell dynamics such as apoptosis in response to environmental stressors (e.g. HIV infection and/or ART). We hypothesized that among individuals with control of HIV replication following initiation of ART, mtDNA variation would influence CD4 count recovery through mechanisms that modulate the efficiency of CD4 cell proliferation, one of these being T cell activation. In ACTG 384, persistent T cell activation was associated with impaired CD4 recovery [17]. To more fully characterize relationships between mitochondrial genomics, T cell activation, and CD4 count recovery, we utilized mtDNA sequence data and comprehensive immunologic data from a subset of ACTG 384 participants.

## Methods

### Ethics Statement

All ACTG 384 sites obtained local institutional review board (IRB) approval, and all study subjects provided written, informed consent. The ACTG Human DNA Repository (HDR) protocol (A5128) underwent separate IRB review at each ACTG site, and subjects provided separate written, informed consent for inclusion of samples in the HDR. The Vanderbilt Committee for the Protection of Human Subjects and the ACTG approved the use of de-identified genetic and clinical data used in these analyses.

### Study Population

ACTG 384 (NCT00000919) was a multicenter, double-blind, prospective randomized clinical trial comparing the efficacy of ART regimens consisting of three or four drugs in antiretroviral-naïve adults [40]–[41]. Participants were randomized to one of six treatment arms consisting of either didanosine and stavudine or zidovudine and lamivudine combined with either efavirenz, nelfinavir of both. A secondary end point of ACTG 384 was change in CD4 cell count from baseline over 48, 96 and 144 weeks. A subset of individuals underwent comprehensive immunologic assessments including proportions of memory (CD45RO+/CD45RA−) and naïve (CD45RA+/CD62L+) CD4 cells and activated (CD38+/HLA-DR+) T cells by flow cytometry [17]. Baseline characteristics for the subjects undergoing comprehensive immunologic assessments were not different than the overall study population, with the exception of inclusion of fewer Hispanic individuals [17]. For the current study, absolute CD4 counts and immunologic T cell assessments at baseline and week 48 from self-reported non-Hispanic black ACTG 384 participants who consented to provide DNA to the ACTG HDR [42] were analyzed. Individuals with HIV-1 RNA ≥400 copies/mL at week 48 were excluded from analyses. Previous publications have reported results from genetic association analysis in the ACTG 384 cohort, and demonstrated no significant differences between ACTG participants who provided DNA as part of the HDR and those who did not [43]–[45].

### Mitochondrial DNA Isolation and Sequencing

DNA was isolated from study participants using PUREGENE (Gentra Systems Inc., Minneapolis, MN, USA). Full mtDNA sequencing was performed using the GeneChip Human Mitochondrial Resequencing Array v2.0 (Affymetrix, Inc., Santa Clara, CA, USA) [46]. Mitochondrial DNA variants were defined by comparison with the revised Cambridge Reference Sequence (rCRS) [47]. Haplogroups were assigned using Herrnstadt classification [48], and collapsed into higher branch haplogroups for analyses.

### Statistical Analysis

The primary outcome of interest was change in percentage CD4 cell activation at week 48. Additional outcomes of interest included baseline and 48 week changes in other CD4 cell immunologic parameters, and CD8 cell counts and activation. Baseline parameters were compared by haplogroups using Wilcoxon ranksum, Fisher’s exact, or chi-squared tests as appropriate. Wilcoxon ranksum test was used to compare distribution of the outcomes by major African mtDNA haplogroups. Multivariate linear regression was used to assess associations between mtDNA haplogroups and CD4 cell activation adjusting for baseline age, absolute CD4 count, HIV RNA, and naïve-to-memory CD4 cell ratio. In the primary and immunologic analyses of ACTG 384 [17], [40], ART regimen was not found to be a significant predictor of CD4 cell recovery following ART initiation and thus was not adjusted for during analysis. Secondary outcomes included an increase of ≥100 CD4 cells/mm^3^ at week 48. In order to maximize power, primary analyses compared non-Hispanic black persons belonging to the L2 major haplogroup with all other non-Hispanic blacks. We also explored differences between L2 and other individual major haplogroups L1 and L3. Analyses were performed using STATA/SE 10.1 (StataCorp, College Station, TX).

## Results

ACTG 384 enrolled a total of 980 participants, 35% of whom were self-identified non-Hispanic black race/ethnicity [40]. Of these, 623 (64%) underwent comprehensive immunophenotyping; 39% of non-Hispanic black race/ethnicity [17]. For initial mtDNA analyses, 423 participants (126 [30%] non-Hispanic black) had mtDNA sequencing that passed all genotyping quality filters, HIV RNA <400 copies/mL at week 48, and CD4 cell counts available [39]. Analyses presented here include 104 of these 126 non-Hispanic black participants with available T cell activation data. Baseline demographics, T cell immunologic assessments, and randomized ART for these subjects are shown in Table 1. The median age was 37 years (range 17, 72). Median baseline CD4 count was 283 cells/mm^3^ (interquartile range [IQR] 111, 453); log_10_ HIV-1 copies/mL was 4.8 (IQR 4.1, 5.4), and percentage activated CD4 and CD8 cells were 15 (IQR 8, 29) and 45 (IQR 33, 58), respectively. Baseline naïve-to-memory cell ratio was available for 91 (88%) subjects, with a median of 0.47 (IQR 0.19, 0.87) among these. CD4 cells correlated with percentage activated CD4 cells at baseline (rho = −0.68; p<0.001) and week 48 (−0.53; p<0.001).

**Table 1:** Baseline demographic data, T cell parameters, HIV RNA, and ART randomization arms among the black, non-Hispanic ACTG 384 population with baseline CD4 activation markers and 48 week HIV RNA <400 copies/mL, total and by African L2 and non-L2 mtDNA haplogroups.

Parameter	Total (N = 104)	L2 (N = 31)	Non-L2 (N = 73)	p-value^a^
Age (years)	37 (32, 46)	35 (31, 43)	39 (32, 46)	0.44
Female sex, N (%)	29 (28)	6 (19)	23 (32)	0.24
CD4+ (cells/mm^3^)	283 (111, 453)	299 (117, 469)	280 (105, 420)	0.63
Naïve CD4+ (cells/mm^3^) [N = 95]^b^	78 (14, 168)	60 (16, 139)	86 (14, 186)	0.47
Memory CD4+ (cells/mm^3^) [N = 91]^b^	154 (53, 238)	154 (97, 352)	154 (41, 217)	0.37
Naïve/memory CD4+ cell [N = 91]^b^	0.47 (0.19, 0.87)	0.41 (0.13, 0.72)	0.48 (0.22, 1.00)	0.32
CD4+ CD38+/DR+ (cells) [N = 95]^b^	31 (15, 46)	25 (15, 40)	34 (17, 51)	0.11
CD4+ CD38+/DR+ (%)	15 (8, 29)	12 (6, 22)	17 (8, 32)	0.03
CD8+ (cells/mm^3^)	777 (471, 1212)	907 (436, 1349)	775 (481, 1101)	0.80
CD8+ CD38+/DR+ (cells) [N = 95]^b^	315 (183, 548)	301 (137, 521)	343 (221, 548)	0.34
CD8+ CD38+/DR+ (%)	45 (33, 58)	41 (26, 54)	47 (35, 59)	0.06
HIV RNA	4.8 (4.1, 5.4)	4.8 (4.1, 5.3)	4.8 (4.0, 5.4)	0.79
Randomized ART, N (%)				
ZDV+3TC^c^	56 (54)	20 (65)	36 (49)	0.20
NFV	17 (30)	6 (30)	11 (31)	
EFV	16 (29)	5 (25)	11 (31)	0.88
NFV+EFV	23 (41)	9 (45)	14 (39)	
ddI+d4T^c^	48 (46)	11 (35)	37 (51)	0.20
NFV	13 (27)	4 (36)	9 (24)	
EFV	16 (33)	6 (55)	10 (29)	0.06
NFV+EFV	19 (40)	1 (9)	18 (49)	

Data are median (IQR) except where noted.

a Wilcoxon ranksum, Fisher’s exact, or Pearson Chi^2^ tests, L2 vs. non-L2;

b N with available baseline data shown;

c p-values for NRTI backbone comparison shown.

Haplogroup distributions (including subhaplogroups) are shown in Table 2. Major African haplogroups included L1 (N = 25 [24%]), L2 (N = 31 [30%]), and L3 (32 [31%]). Non-African haplogroups were observed in 14 (13%) individuals included in analyses of non-L2 comparison groups. Secondary analyses limited to persons having African haplogroups yielded similar results (data not shown). Baseline age, absolute CD4 count, and plasma HIV RNA were not statistically different between haplogroup L2 individuals and others (Table 1). Randomized NRTIs did not differ statistically by haplogroup (p = 0.20), but L2 individuals randomized to didanosine plus stavudine tended to be randomized to the efavirenz arm more often than non-L2 individuals (p = 0.06).

**Table 2:** Mitochondrial DNA haplogroup frequencies among black, non-Hispanic ACTG 384 participants.

Haplogroup/Subhaplogroup- N (%)^a^	Total non-Hispanic black with mtDNA sequence data (N = 126) [39]	Non-Hispanic black with mtDNA sequence and baseline CD4+ activation data (N = 104)
African Haplogroups		
L1	31 (25)	25 (24)
L1a	7 (6)	4 (4)
L1b	10 (8)	9 (9)
L1c	14 (11)	12 (12)
L1/L2	2 (2)	2 (2)
L2	40 (32)	31 (30)
L2a	25 (20)	22 (21)
L2b	15 (12)	9 (9)
L3	36 (29)^b^	32 (31)^b^
L3b	12 (10)	11 (11)
L3e	8 (6)	7 (7)
Non-African Haplogroups		
A	4 (3)	3 (3)
H2	4 (3)	4 (4)
Others^c^	9 (7)	7 (7)

a Totals may not  = 100% due to rounding;

b Total includes haplogroup L3, not sub-haplogrouped;

c Includes haplogroups H, I, J1, T1, T2b, U6, U9 (N≤2 each).

**Figure 1:**
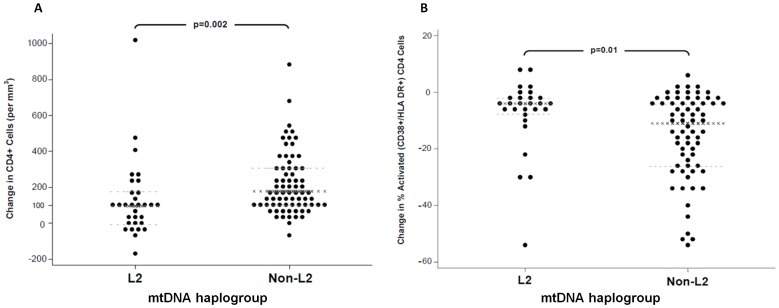
Scatter plots of 48 week changes in CD4 cells (Panel A), and percentage activated (CD38+/HLA-DR+) CD4 cells (Panel B). Haplogroup L2 individuals had a significantly lower median increase in CD4 cells and a lower decrease in percentage activated CD4 cells. Unadjusted p-values by Wilcoxon ranksum test shown. x-lines  = median; dashed lines = 25^th^ and 75^th^ percentiles.

When comparing mtDNA haplogroup L2 with all other haplogroups (Table 1), baseline median percentage activated (CD38+/HLA-DR+) CD4 cells was significantly lower (12% [IQR 6, 22] vs. 17% [8], [32]; p = 0.03) in persons belonging to the L2 haplogroup. Median absolute activated CD4 cells was lower (25 [15], [40] vs. 34 [17], [51] cells/mm^3^; p = 0.11), but was not statistically different. Median percentage activated CD8 cells also tended to be lower (41% [26], [54] vs. 47% [35], [59]; p = 0.06). After 48 weeks of ART, median CD4 cells was not statistically different in the L2 versus non-L2 haplogroups (399 [IQR 168, 670] vs. 472 [292, 732]; p = 0.13; Table 3). However, the median change from baseline was significantly less among L2 versus non-L2 haplogroups (+95 [−3, +182] cells vs. +178 [+105, +312]; p = 0.002; Figure 1A). Similarly, although 48 week percentage activated CD4 cells did not differ (7% [4], [11] vs. 6% [4], [9]; p = 0.57), the median change in percentage activated CD4 cells was significantly less in the L2 compared with the non-L2 haplogroups (−4% [−8, −2] vs. −11% [−26, −4]; p = 0.01; Figure 1B). Correlations between CD4 cells and percentage activated CD4 cells at baseline and week 48 were similar among L2 and non-L2 haplogroups (data not shown).

**Table 3:** T cell parameters at 48 weeks and 48 week changes among the black, non-Hispanic ACTG 384 population with baseline CD4 activation markers and 48 week HIV RNA <400 copies/mL, total and by African L2 and non-L2 mtDNA haplogroups.

Parameter	Total non-Hispanic black (N = 104)	L2 (N = 31)		Non-L2 (N = 73)		p-value^a^
**Week 48**						
CD4+ (cells/mm^3^)	460 (230, 711)	399 (168, 670)		472 (292, 732)		0.13
Naïve CD4+ (cells/mm^3^) [N = 98]^b^	148 (70, 323)	96 (48, 253)		165 (79, 346)		0.06
Memory CD4+ (cells/mm^3^) [N = 98]^b^	247 (133, 326)	228 (113, 359)		251 (134, 317)		0.89
CD4+ CD38+/DR+ (cells) [N = 97]^b^	24 (15, 43)	20 (12, 37)		24 (17, 44)		0.17
CD4+ CD38+/DR+ (%) [N = 97]^b^	6 (4, 10)	7 (4, 11)		6 (4, 9)		0.57
CD8+ (cells/mm3)	812 (554, 1031)	726 (470–914)		855 (575, 1072)		0.07
CD8+ CD38+/DR+ (cells) [N = 99]^b^	127 (88, 252)	115 (90, 182)		141 (88, 273)		0.34
CD8+ CD38+/DR+ (%) [N = 112]^b^	21 (13, 29)	20 (14, 28)		21 (11, 30)		0.87
**Week 48-Week 0**						
CD4+ change (cells/mm^3^)	+135 (+79, +271)		+95 (−3, +182)		+178 (+105,+312)	0.002
N (%) with ≥100 CD4 cell increase	68 (65)		13 (42)		55 (75)	0.002
Naïve CD4+ change (cells) [N = 90]^b^	+74 (+29, +161)		+43 (+20, +80)		+87 (+31, +171)	0.06
Memory CD4+ change (cells) [N = 86]^b^	+82 (+30, +135)		+50 (+15, +125)		+104 (+35, +139)	0.11
CD4+ CD38+/DR+ change (cells) [N = 89]^b^	−2.3 (−18, +10)		−4.5 (−11.8, +13.7)		−2.3 (−23.6, +7.5)	0.37
CD4+ CD38+/DR+ change (%) [N = 97]^b^	−8 (−22, −3)		−4 (−8, −2)		−11 (−26, −4)	0.01
CD8+ change (cells/mm3)	−67 (−320, +223)		−148 (−540, +116)		−61 (−255, +260)	0.17
CD8+ CD38+/DR+ change (cells) [N = 90]^b^	−188 (−324, −51)		−143 (−307, −48)		−204 (−333, −60)	0.46
CD8+ CD38+/DR+ change (%) [N = 99]^b^	−26 (−33, −13)		−17 (−33, −10)		−27 (−33, −17)	0.09

Data are median (IQR) except where noted.

a Wilcoxon ranksum, Fisher’s exact, or Pearson Chi^2^ tests, L2 vs. non-L2;

b N with available data shown.

Among other T cell measures, median naïve CD4 cells (p = 0.06) and absolute CD8 cell counts (p = 0.07) at 48 weeks both tended to be lower among individuals belonging to the L2 haplogroup (Table 3). Median week 48 increases in naïve CD4 cells also tended to be lower (+43 [+20, +80] vs. +87 [+31, +171]; p = 0.06) among haplogroup L2 individuals, as were decreases in activated CD8 cells (−17% [−33, −10]; −27 [−33, −17]; p = 0.09), but these differences were not statistically significant. As observed in our earlier study, [39] persons in this sub-analysis belonging to haplogroup L2 were significantly less likely to have an increase of ≥100 CD4 cells over the first 48 weeks of suppressive ART compared to non-L2 individuals (42% vs. 75%; p = 0.002).

In separate multivariate models, associations between mtDNA haplogroup L2 and baseline (pre-ART) and 48-week changes in percentage activated CD4 cells were assessed with adjustment for baseline age, absolute CD4 count, HIV RNA, and naïve-to-memory CD4 cell ratio. [17] In both models, baseline CD4 count was strongly associated with percentage activated CD4 cells (p<0.001; Table 4). Haplogroup L2 was also associated with lower baseline (β = −6.7 [95% CI −12.8, −0.50]; p = 0.035) and smaller 48-week change (β = 7.5 [1.6,13.5]; p = 0.013) in percentage activated CD4 cells.

**Table 4:** Multivariate models of associations between baseline covariates, haplogroup L2, and percentage CD4 cell activation.

	Model 1: Baseline % activated CD4 cells (N = 91)		Model 2: 48 week change in % activated CD4 cells (N = 85)	
Covariates	β (95% CI)	p-value	β (95% CI)	p-value
Age (per year increase)	−0.02 (−0.29, +0.25)	0.88	+0.10 (−0.15, +0.36)	0.42
Baseline plasma HIV RNA (per log_10_ copies/mL increase)	+2.04 (−1.53, +5.61)	0.26	−2.11 (−5.46, +1.23)	0.21
Naïve-to-memory CD4 cell ratio (per unit increase)	−0.46 (−1.40, +0.48)	0.33	+0.24 (−0.62, +1.10)	0.58
Baseline CD4 cells (per 10 cell increase)	−0.40 (−0.54, −0.25)	<0.001	+0.27 (+0.13, +0.40)	<0.001
Haplogroup L2 (vs. others)	−6.67 (−12.84, −0.50)	0.035	+7.54 (+1.62, +13.46)	0.013

## Discussion

This analysis expands a previous study of mitochondrial genomic predictors of CD4 cell recovery in ACTG Study 384 [39]. Differences in T cell subsets and activation markers were observed within the African mtDNA haplogroup that also demonstrated impaired CD4 cell recovery despite suppressed HIV RNA after 48 weeks on ART. The most notable differences included lower CD4 cell activation at baseline despite similar absolute CD4 counts and HIV RNA, and less decrease in percentage activated CD4 cells together with a less robust increase in absolute CD4 cells after 48 weeks of ART (as was previously reported [39]). There were also several intriguing trends- including in naïve CD4 cell populations, and absolute and activated CD8 cell subsets- that did not reach statistical significance in this small sample but deserve additional study. Although replication in larger studies and mechanistic validation are needed to confirm these findings, we interpret these results as suggesting that mtDNA variation among these non-Hispanic black clinical trial participants influenced the magnitude of CD4 cell recovery in the first year after ART by modulating T cell activation through as-yet-unknown mechanisms. This modulation was independent of other baseline predictors of short-term CD4 recovery, including age, CD4 count, and naïve-to-memory CD4 cell ratio.

The role of the mitochondrion as an apoptotic regulator is well recognized, and its specific relevance in T cells during HIV-infection has been studied [49]. In a recent study, peripheral blood mononuclear cells (PBMCs) from HIV-infected long-term non-progressors demonstrated less mitochondrial dysfunction and mitochondria-mediated apoptosis than typical progressors [50]. Studies have also observed reduced mtDNA quantity in PBMCs from ART-naïve, HIV-infected compared with HIV-negative individuals [37], [51]. One study reported decreased mtDNA that correlated with increased T cell activation in chronically infected, untreated men [36]. Another found decreased mitochondrial membrane potential and increased apoptosis in PBMCs from HIV-infected, ART-naive compared with uninfected subjects [52]–[53]. These measures correlated with each other, and lower mitochondrial membrane potential correlated with lower CD4 T cell counts. In black South Africans [54], total T cell mitochondrial depolarization and CD4 T cell apoptosis were increased in ART-naive compared with treated subjects, and a positive correlation between these parameters was observed among treated subjects, suggesting relationships between mitochondrial function and T cell apoptosis in patients. Finally, recent work from one group has characterized mechanisms by which mitochondria might regulate activation in Jurkat T cell lines in the absence of HIV infection [55]–[56].Taken together, these data strongly suggest that adverse mitochondrial phenotypes prior to ART can contribute to peripheral T cell activation and apoptosis. Published data on the direct effects of mtDNA variation on cellular apoptosis are still limited, but include a murine model of mtDNA depletion [57], and a small study of patients with mtDNA tRNA point mutations showing an increase in apoptotic muscle fibers (by TUNEL staining) [58]. An *in vitro* study in fibroblasts demonstrated massive ROS-induced apoptosis in the presence of a mtDNA point mutation [59].

Based on this emerging literature and our results, one could speculate regarding several possible mechanisms by which mtDNA variants (in this case, those specifically marking the L2 haplogroup) might influence CD4 cell recovery. First, T cells harboring specific L2-associated mtDNA variants may have differential ability to become and/or remain activated in the presence of antigenic stimulation. Second, mtDNA variation could lead to differential susceptibility to cell death (via the intrinsic apoptotic pathway) in the setting of chronic T cell activation prior to suppression of HIV replication with ART. This could lead to a lower percentage of activated T cells prior to starting ART, as was observed here. We also observed a less robust decrease in percentage activated CD4 (and to a lesser extent, CD8) cells during the first 48 weeks of ART. If a greater proportion of activated cells progress to apoptotic cell death among persons with haplogroup L2, this could explain a less robust gain in absolute CD4 cells over the same time period. This less robust CD4 cell increase may also be related to fewer numbers of naïve CD4 cells- an important component of CD4 recovery after suppression of viral replication in this population [17], [22]- both at baseline and after 48 weeks of ART.

CD4 cell activation has been associated with CD4 cell recovery [15]–[16]. In the overall immunologic analysis of this population [17], there was no correlation between baseline CD4 cell activation and CD4 recovery on ART, but higher CD4 and CD8 activation at week 48 correlated with CD4 recovery at week 48. Although the direction of associations were similar in our subgroup, we did not observe significant correlations between 48-week CD4 or CD8 activation and CD4 recovery (data not shown), perhaps due to the smaller sample size. However, absolute CD4 cells were strongly correlated with percentage activated CD4 cells at both time points.

A recent analysis using data from several U.S. cohorts reported associations between European haplogroups and increased prevalence of pre-ART progression to AIDS and/or CD4 count <200 cells/mm^3^ among Caucasians [38]. These findings are also consistent with a role for mtDNA variation in CD4 T cell dynamics. Another study did not find cross-sectional associations between mtDNA haplogroups and current CD4 counts or viral load among a population of predominantly ART-treated HIV-infected Italians [60]. The present study is, to our knowledge, the first to assess relationships between *ex vivo* measures of T cell activation and mitochondrial genomic variation in a population with longitudinal data from before and after ART.

Limitations of our data include a small sample size which may have impaired our ability to detect less robust associations. We elected to use self-identified race/ethnicity to define our analysis group instead of genetic ancestry. Secondary analyses that included only non-Hispanic black individuals with an African L haplogroup yielded similar results (data not shown). Because of the focused nature of these analyses, results are not generalizable to other racial/ethnic groups. We defined virologic suppression as HIV RNA <400 copies/mL; it is possible that lower levels of viral replication influenced T cell activation in these subjects. However, plasma viral load at week 48 (median [IQR] 1.38 [1.23–1.53] log_10_ copies/mL) was not correlated with either CD4 or CD8 activation at week 48 or 48-week changes in activation (data not shown). Additionally, in a secondary analysis of 89 (86%) persons with 48-week HIV RNA <50 copies/mL, results were also similar (data not shown). All subjects included in these analyses received older NRTI combinations: stavudine plus didanosine, or zidovudine plus lamivudine. Given the lack of association of CD4 cell recovery with study arm in prior analyses of this population [17], [22], [40]–[41], we did not include ART in adjusted models, and do not believe observed relationships between mtDNA haplogroup and T cell activation markers are explained by NRTI mitochondrial toxicity, *per se*. Studies using data from cohorts of subjects exposed to newer NRTIs will be necessary to confirm that relationships persist and are of similar magnitude in this setting. Less robust CD4 cell recovery on ART has been associated with HIV-related disease progression [2], [4], and with incidence of non-AIDS-defining cancers in a cohort study that included some ACTG 384 participants [61]. Our analyses did not include these outcome data, thus we cannot determine whether less robust decreases in CD4 cell activation markers and/or CD4 cell increases observed with the African L2 haplogroup over the first 48 weeks of suppressive ART impacted long-term clinical outcomes. Lastly, although these results suggest that mtDNA variation may influence T cell activation, we do not have direct functional measures of mitochondrial oxidative phosphorylation or apoptosis in these subjects to provide additional data on potential mechanisms. Future studies will include such measures.

In summary, we report what is to our knowledge the first association between mtDNA variation and measures of T cell activation in an HIV infected population. The careful data and specimen collection as part of a prospective clinical trial is a strength of these analyses. Replication of these results in independent clinical trial datasets is needed, ideally including African populations where the L2 haplogroup is prevalent. Larger studies may also allow for more specific assessment of mtDNA subhaplogroups and/or SNPs associated with even more pronounced differences in CD4 recovery. Mechanistic studies of mitochondrial function within T cells are feasible, and will be necessary to further confirm and characterize relationships between mtDNA variants and T cell function. Ultimately, an improved understanding of the role of mitochondria and mtDNA variation in T cell dynamics could lead to improved decisions about when and in whom to initiate ART, and to targeted therapeutic interventions that could augment CD4 recovery and its health benefits in HIV-infected persons.
